# Masseter muscle thickness and vertical cephalometric characteristics in children with Class II malocclusion

**DOI:** 10.1002/cre2.528

**Published:** 2022-02-11

**Authors:** Eirini Tentolouri, Gregory S. Antonarakis, Ioanna Georgiakaki, Stavros Kiliaridis

**Affiliations:** ^1^ Division of Orthodontics University Clinics of Dental Medicine, University of Geneva Geneva Switzerland; ^2^ Private Practice Thessaloniki Greece; ^3^ Department of Orthodontics and Dentofacial Orthopedics University of Bern Bern Switzerland

**Keywords:** children, Class II malocclusion, masseter muscle, vertical characteristics

## Abstract

**Background:**

Masseter muscle thickness and its relationship with vertical craniofacial morphology have been extensively studied in adults, but data on children are lacking.

**Objective:**

To examine the association between masseter muscle thickness and vertical cephalometric parameters in a group of Class II malocclusion growing children.

**Methods:**

The current study design was retrospective and cross‐sectional, looking at a sample of 211 growing children with Class II malocclusion between the ages of 6 and 15 derived from two centers. Ultrasonographic masseter muscle thickness measurements and vertical cephalometric variables, including the gonial angle, were evaluated before any orthodontic treatment had been carried out. Multiple linear regression analysis was used to examine the association between masseter muscle thickness and vertical cephalometric measurements, including age and patient origin as independent variables in the analysis.

**Results:**

In the present sample, masseter muscle thickness was found to be independent of sex, but correlated with age, with older children presenting thicker masseter muscles. In the total patient sample, using multiple regression analyses, children with thicker masseter muscles had significantly smaller intermaxillary and gonial angles. No other cephalometric vertical characteristics showed associations with masseter muscle thickness.

**Conclusion:**

In growing children with Class II malocclusion, those with thicker masseter muscles are more likely to display smaller intermaxillary and gonial angles respectively.

## 1 INTRODUCTION

Different therapeutic approaches for the correction of Class II malocclusion in growing individuals exist, with functional appliances being a widely used means of treatment with potentially successful yet varying results. Functional appliances act on the sagittal and vertical position of the mandible, bringing about both skeletal and dentoalveolar effects. Their skeletal effect is through the displacement of the mandible downwards and forwards which causes soft tissue and muscle stretching and myotatic reflexes (Bishara & Ziaja, 1989; Carels & van der Linden, 1987; Graber & Neumann, 1984). The dentoalveolar effect arises from the shift in the dental arches towards a Class I molar relationship and incisor compensation (Macey‐Dare & Nixon, 1999; Vargervik & Harvold, 1985).

The large variation in interindividual response to functional appliances may be due to several factors including compliance, appliance choice, growth potential, facial type, treatment timing, and skeletal maturity (Barton & Cook, 1997; Bishara & Ziaja, 1989; Carels & van der Linden, 1987; Celli et al., 2010; Tulloch et al., 1990; Woodside, 1998). Various cephalometric characteristics have been proposed to correlate to a favorable response to functional appliance treatment such as a low mandibular plane angle, low basal‐plane angle, high Jarabak ratio, short mandibular corpus and ramus height, short cranial base, and small anterior and posterior lower face heights (Kumar et al., 2013; Patel et al., 2002).

Masticatory muscles, which are directly involved in functional appliance treatment, have also been thought to play an important role in defining the degree of success of this treatment. In fact, recent studies show that the initial condition of the masticatory muscles evaluated either through ultrasonographic masseter muscle thickness measurements or through maximal molar bite force measurements may partly determine functional appliance treatment outcomes (Antonarakis & Kiliaridis, 2015; Antonarakis et al., 2012; Kiliaridis et al., 2010). Moreover, functional appliance treatment seems to also have an effect on the masticatory muscles, as witnessed by temporary atrophy of masseter muscles following functional treatment (Kiliaridis et al., 2010).

In an important study that tried to ascertain what pretreatment cephalometric variables may be predictive of individual mandibular outcomes following functional appliance treatment in children with Class II malocclusion, the discriminant analysis identified a single predictive variable, namely the gonial angle (Franchi & Baccetti, 2006). The authors go on to suggest that children with a pretreatment gonial angle of smaller than 125.5° are expected to respond favorably (a greater skeletal mandibular effect) to functional appliance treatment while those with a gonial angle greater than 125.5° will be expected to respond unfavorably. More recent studies (Antonarakis & Kiliaridis, 2015; Kiliaridis et al., 2010) also find associations between the gonial angle and treatment outcome following functional appliance treatment, which agree with the results previously mentioned. A study on children treated with twin blocks found that the only predictive variable for successful treatment was the condylion–gonion–menton angle (Cretella Lombardo et al., 2020). Based on these data, it has thus been suggested that the gonial angle may be able to be used as a proxy for the functional capacity of the masticatory muscles, however, the samples in these previous studies were relatively small.

Masticatory muscle function is related to increased loading of the jaws and bone apposition which can have an effect on the gonial angle (Kiliaridis, 2006). The gonial angle is a site of muscle attachment (masseter and medial pterygoid muscles) and thus the size and activity of these muscles may affect the morphology of the gonial angle, and perhaps the dentofacial morphology more generally. Wolff's law explains that bone morphology is affected by muscle thickness (Wolff, 1870). This means that there is an association between the function of muscles and the internal structure and shape of the bone (Dibbets, 1992). Putting this hypothesis into practice, research suggests that there is a negative association between masseter muscle thickness and vertical facial morphology (Hannam & Wood, 1989; Kiliaridis & Kalebo, 1991; Raadsheer et al., 1996; Weijs & Hillen, 1986). All of these cited studies however investigate adult samples, besides one (Raadsheer et al., 1996). That one study however looked at a heterogeneous group of individuals, including both children and adults, from 7 to 22 years of age and evaluated anthropometric instead of cephalometric data.

Although of fundamental interest in helping us understand variation in the response to functional appliance treatment, the direct influence of the masticatory muscles on treatment outcome does not have everyday clinical applicability as orthodontic offices are very seldomly equipped with an ultrasound machine or a bite force measuring gauge. Finding a clinically useful variable, such as the gonial angle, that can act as a proxy for the masticatory muscle functional capacity would therefore be very useful in a clinical setting, as this may aid in treatment planning or more accurate prediction of treatment outcome.

The aim of our study was thus to investigate the association between masseter muscle thickness and vertical cephalometric variables, including the gonial angle, in a homogeneous group of growing children with Class II malocclusion.

## 2 MATERIALS AND METHODS

### 2.1 Subjects

The present retrospective cross‐sectional study was performed on a sample of 211 growing children with Class II malocclusion (115 females and  96 males) between the ages of 6 and 15, from two different centers. The patients were seen either at our University Orthodontic Clinic (*n* = 82) or in a private practice limited to orthodontics (*n* = 129). The study was approved by the Cantonal Research Ethics Commission (no. 2016‐00292), and informed consent was not applicable since the data were collected retrospectively.

The inclusion criteria were the following: children with Class II malocclusion presenting for orthodontic treatment, where pretreatment records had been taken; the presence of a pretreatment cephalometric radiograph; ultrasonographic masseter muscle thickness measurements having been undertaken.

The exclusion criteria were the following: patients without the presence of the first permanent molars; patients with any craniofacial anomaly or syndrome; juvenile idiopathic arthritis or signs of condylar lesions or temporomandibular dysfunction or disorders; lateral cephalometric radiographs of insufficient diagnostic quality; insufficient data with regard to the masseter muscle thickness measurements (not done bilaterally, or not done in contraction).

### 2.2 Methods

The two principal outcomes evaluated were pretreatment masseter muscle thickness evaluated by ultrasonographic measurements, and pretreatment cephalometric analysis focusing on the vertical dimension.

#### 2.2.1 Masseter muscle thickness measurements

The thickness of the masseter muscles was measured using ultrasonography, as per the method described by Kiliaridis and Kälebo (Kiliaridis & Kalebo, 1991), and modified by Raadsheer et al. (1994). Children involved in our study were examined by one of two operators (G. S. A. or I. G.), in either of the two centers, using a real‐time scanner (Pie Medical Scanner 480) with a 7.5 MHz linear array transducer. The two operators had been calibrated to the senior author having originally developed the method (S. K.). Imaging and measurements were performed bilaterally with the subjects seated in an upright position, with their heads in a natural head position without a headrest. Measurements were taken with the muscles in contraction. Children were asked to clench maximally in the intercuspal position so that the masseter muscles were contracted. Scanning of the masseter muscle was then performed on a level halfway between the zygomatic arch and the gonial angle. To avoid erroneous measurements, the scan plane was orientated perpendicular to the anterior border of the muscle and to the surface of the underlying ramus, and light pressure was applied to the muscles. The registrations were performed twice for each muscle, and the final muscle thickness was calculated as a mean of the duplicate measurements of the contracted muscles. The measurements were taken directly from the image at the time of scanning with a read‐out distance to the nearest 0.1 mm.

#### 2.2.2 Cephalometric analysis

**Figure 1:**
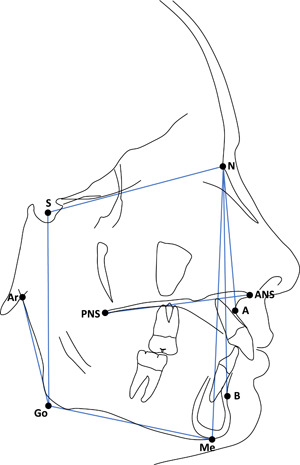
Cephalometric measurements used: ANB angle—the angle formed between points A, nasion (N), and B; intermaxillary angle—the angle formed between the maxillary and mandibular planes, with the maxillary plane being defined as a plane between the anterior nasal spine (ANS) and posterior nasal spines (PNS), and the mandibular plane being defined as a plane between menton (Me) and gonion (Go); mandibular plane angle—the angle formed between the Sella‐Nasion (SN) plane, and the MeGo plane; gonial angle—the angle formed between articulare (Ar), Go, and Me; mandibular ramus height—the distance between Ar and Go; facial height index—ratio of posterior facial height (PFH, defined by S‐Go) to anterior face height (AFH, defined by N‐Me)

All lateral cephalometric radiographs were carried out in a standardized way, with the head fixed in a cephalostat and with the teeth in occlusion. One operator (E. T.) performed the cephalometric analysis of all radiographs using OnyxCeph^3TM^ (Image Instruments). The magnification of all radiographs was adjusted to zero. A limited cephalometric analysis was carried out; the measurements that were used are shown in Figure 1.

### 2.3 Statistics

The statistical analysis of our data was performed using the Statistical Package for Social Sciences version 25.0 (SPSS Inc). First, sex, age, and the ANB angle were tested for possible associations with masseter muscle thickness. Linear regression analysis was then used to study the association between masseter muscle thickness and the vertical cephalometric measurements, for the total patient sample. Finally, multiple regression analyses were performed including age, sex, patient origin (from which center), and/or the ANB angle as additional independent variables and masseter muscle thickness as the dependent variable. All correlations were considered significant at *p* < .05.

### 2.4 Error of the method

Paired *t*‐tests were used to calculate the systematic error and Dahlberg's formula to measure random error (Houston, 1983), for both ultrasonographic masseter muscle thickness measurements and the cephalometric analysis. The error of the method for the masseter muscle thickness measurements was carried out by the two investigators having carried out these measurements. Each investigator performed repeated measurements on twenty patients on two separate occasions, 2–4 weeks apart. No systematic error was found, and random error was found to be 0.3 mm for one of the investigators and 0.4 mm for the other investigator. The error of the method for the cephalometric analysis was calculated by repeating cephalometric tracings on 20 radiographs, with a 2‐week interval separating the two measurements. No significant systematic error was found, and random error was found not to exceed 1° for angular measurements and 0.9 mm for linear measurements.

## 3 RESULTS

### 3.1 Baseline data

The subjects involved in this study consisted of 211 children (115 females and 96 males) with Class II division 1 malocclusion, between the age of 6 and 15 (mean age: 10.3, standard deviation [SD]: 1.9 years). A total of 129 of the patients originated from one center, and 82 from the other center. The sample was collected based on a dental Class II malocclusion, and the mean skeletal anteroposterior relationships (as measured with the ANB angle) was 5.3 (SD: 1.8) degrees. The mean masseter muscle thickness, measured under contraction, was found to be 11.7 (SD: 1.7) mm in the present sample.

Baseline data for the sample are presented in Table 1. No statistically significant differences were found between females and males for any of the variables examined.

**Table 1:** Baseline data for the sample of Class II malocclusion children

	Total sample (*n* = 211)		Females (*n* = 115)		Males (*n* = 96)	
	Mean	SD	Mean	SD	Mean	SD
Age (years)	10.3	1.9	10.3	1.9	10.4	1.9
Masseter muscle thickness (mm)	11.7	1.7	11.5	1.7	11.9	1.6
ANB (degrees)	5.3	1.8	5.2	1.9	5.3	1.6
Intermaxillary angle (degrees)	28.1	5.4	28.4	5.4	27.8	5.3
Mandibular plane angle (degrees)	34.9	5.4	35.5	5.5	34.2	5.2
Gonial angle (degrees)	126.3	5.9	126.6	6.0	125.9	5.9
Mandibular ramus height (mm)	42.9	8.7	42.2	8.7	43.8	8.8
Facial height ratio (%)	63.5	4.6	63.2	4.6	64.0	4.8

### 3.2 Masseter muscle thickness

**Figure 2:**
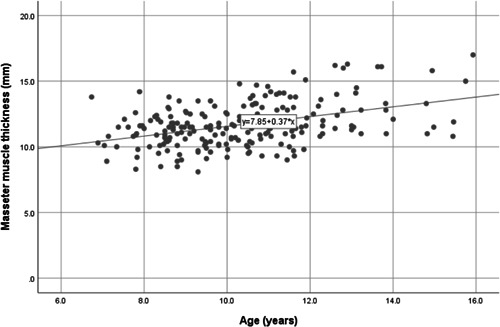
Scatter plot showing the correlation between masseter muscle thickness and age (*R* = .419, *p* < .001)

Masseter muscle thickness in the present sample was independent of sex but correlated with age (*R* = .419, *p* < .001), with older children having thicker masseter muscles. The scatter plot in Figure 2 shows the correlation between masseter muscle thickness and age. No association was found between the ANB angle and masseter muscle thickness. When performing a regression model with masseter muscle thickness as the dependent variable and age, sex, and patient origin as independent variables, the model is statistically significant (*R* = .437, *p* < .001) with only age being significant within the model (*β* coefficient = .403, *p* < .001).

### 3.3 Masseter muscle thickness and vertical cephalometric variables

Using simple bivariate regression analysis, several of the cephalometric variables measured showed statistically significant correlations with masseter muscle thickness. There were significant, although weak, correlations of masseter muscle thickness with the intermaxillary angle (*R* = .166, *p* = .026), the mandibular plane angle (*R* = .198, *p* = .016), the gonial angle (*R* = .185, *p* = .008), and the facial height ratio (*R* = .167, *p* = .043). Thicker masseter muscles were seen in children with a smaller intermaxillary and mandibular plane angle, a smaller gonial angle, and a smaller facial height ratio.

When performing multiple regression analyses, age, sex, and center were added as additional independent variables to the regression models. Analyses show significant correlations with the gonial angle and the intermaxillary angle, both of which showed a negative correlation with masseter muscle thickness. The results of these multiple regression analyses are shown in Table 2.

**Table 2:** Multiple linear regression analysis results with the cephalometric variable as the dependent variable

	*R*	Significance of model	*β* Coefficient	Significance of variable
Model 1—gonial angle	.362	*p* < .001		
Constant			133.8	*p* < .001
Masseter muscle thickness (mm)			−.8	*p* = .003
Age (years)			−.2	*p* = .491
Sex (0 = female and 1 = male)			−.2	*p* = .785
Patient origin (Center 1 or 2)			2.8	*p* = .001
Model 2—intermaxillary angle	.298	*p* = .001		
Constant			39.9	*p* < .001
Masseter muscle thickness (mm)			−0.8	*p* = .001
Age (years)			−0.2	*p* = .401
Sex (0 = female and 1 = male)			−0.5	*p* = .481
Patient origin (Center 1 or 2)			0.1	*p* = .892

*Note*: With regard to patient origin, Center 1 represents the private practice while Center 2 represents the University Orthodontic Clinic.

## 4 DISCUSSION

The results of the present study show associations between the thickness of the masseter muscle and the intermaxillary and gonial angles in a sample of growing children with Class II malocclusion. More specifically, in children with thicker masseter muscles, a smaller intermaxillary and gonial angle were found.

Previous studies, although having been carried out in adults, have provided results which point in the same direction, with numerous studies (Bakke et al., 1992; Benington et al., 1999; Farella et al., 2003; Kiliaridis & Kalebo, 1991; Raadsheer et al., 1996; Rani & Ravi, 2010; Satiroğlu et al., 2005; Weijs & Hillen, 1984) finding a negative association between masseter muscle thickness and facial morphology, and vertical facial height. Cephalometric variables such as the mandibular plane angle and the gonial angle have been negatively associated with masseter muscle thickness, and mandibular ramus height and posterior facial height have been positively associated with masseter muscle thickness (Kubota et al., 1998; Rohila et al., 2012).

Other methods to measure the functional capacity of the masticatory muscles have also been looked at in relation to the vertical facial morphology, often arriving at similar conclusions. Vertical facial dimensions have been found to be negatively correlated with maximal bite force and the electromyographic activity of the masticatory muscles (Custodio et al., 2011; Takeuchi‐Sato et al., 2019). Computer tomography (CT) studies have also found comparable results with masseter muscle thickness and length showing a negative correlation with the mandibular plane angle (Azaroual et al., 2014).

The majority of the data available however to date are derived from adult samples. Few studies have looked at associations between the functional capacity of the masticatory muscles and vertical craniofacial morphology in growing children. In a sample of 60 prepubertal children, Lione et al. (2013) found that the masseter muscles presented a significantly decreased ultrasonographic volume in dolichofacial subjects compared with brachyfacial or normofacial subjects by looking at the mandibular plane angle. In a study on 61 growing children, Biondi et al. (2016) showed that masseter muscle thickness was progressively decreased in low‐angle, normal‐angle, and high‐angle subjects, with the vertical skeletal pattern being evaluated using the Frankfurt‐mandibular plane angle. Another study carried out on 72 children between 8.5 and 9.5 years of age found that the importance of the masseter muscle is more evident in the vertical facial morphology of females, whereby there was a negative association between masseter muscle thickness and the intermaxillary angle (Charalampidou et al., 2008).

Using other methods to evaluate the functional capacity of the masticatory muscles, comparable results have also been obtained. Ingervall and Thilander (1974) showed that children with greater muscle activity during maximal contraction presented a more rectangular facial type. However, one study in growing children looking at maximal bite force found correlations between bite force and craniofacial dimensions only for boys (Sonnesen & Bakke, 2005).

Using three‐dimensional CT imaging in growing children, it has been found that in dolichofacial subjects with an increased mandibular plane angle, a decreased posterior facial height, and a decreased facial height ratio, the angle between the anterior border of the masseter and the Frankfurt horizontal plane is likely to be considerably less than in brachyfacial subjects (Chan et al., 2008; Wong et al., 2016).

It is unclear at what point during growth and development the associations between the masticatory muscles and vertical facial morphology become more evident, or what the exact cause and effect is. Proffit and Fields (1983) suggested that low bite force in subjects with a hyperdivergent facial pattern might allow excessive eruption of posterior teeth and backward rotation of the mandible although they were unable to find any significant differences between normal‐ and long‐face children with regard to bite force. This explanation is also offered by other authors, although based on a rat experimental model, agreeing that the hypofunction of the muscles in a hyperdivergent pattern produces weaker forces, and this results in a greater eruption of posterior molars and more vertical growth (Bresin, 2001).

The originality in the present study is that associations between masseter muscle thickness and vertical craniofacial parameters are investigated in a large homogeneous sample of children only with Class II malocclusion. One must keep in mind that no sex differences were found in the present sample probably because children were less than 15 years of age, and sex differences normally appear after puberty and into adulthood.

Data resulting from the present study can provide clinical relevance with regard to the treatment of Class II malocclusion in growing children with the use of functional appliances. The gonial angle is a site of muscle attachment and could therefore be used as a representative variable defining the masticatory system. A large gonial angle may indicate that the masticatory system is weaker and a smaller gonial angle that there is a better developed masticatory system. As this is easily measurable in any lateral cephalometric radiograph, it could be looked at in all children to evaluate the masticatory system before commencing functional appliance treatment to be able to partly predict treatment outcomes.

Muscular factors in relation to vertical craniofacial morphology are one of many factors that can play an important role in dictating this morphology. Particularly in children who are in their mixed dentition, the number of interocclusal contacts during the period of the transition of the dentition may be responsible for some of the variation in muscle thickness measurements, which may attenuate any association between muscles and craniofacial morphology. Occlusal contacts have been found to be associated with muscle thickness and function (Bakke et al., 1992; Ferrario et al., 2002). There are of course other variables such as function, genetics, or ethnic origin which may be equally or more important for craniofacial growth. A complex interaction between genetics and environmental influences can determine the development of craniofacial morphology during growth.

### 4.1 Limitations

The present study evaluated a large sample of children, more than 200, and it is thus to date the largest study attempting to look at associations between masseter muscle thickness and vertical craniofacial morphology in growing children. Limitations however include that its design is retrospective and cross‐sectional in nature. Ideally, a prospective longitudinal study would be able to give more concrete answers with regard to the development of the vertical craniofacial morphology in children in relation to their masticatory functional capacity.

A further possible limitation is the fact that the study was carried out in a homogeneous sample, looking specifically at children with Class II malocclusion, which raises questions about generalizability to other malocclusions or populations. The advantage of including only children with Class II malocclusion however is to ensure a certain homogeneity and clinical relevance in respect to treatment outcomes in this group of children such as with functional appliances. Finally, the patients are derived from two different centers with differences in the populations, and the operator carrying out the ultrasonographic masseter muscle thickness measurements. Interoperator reliability was not carried out since measurements were not carried out on the same patients by the two examiners, but both examiners were nevertheless calibrated to the senior author. Moreover, the center of origin was included as an independent variable in multiple regression analyses in an effort to account for these differences and possible systematic error.

## 5 CONCLUSIONS

- Masseter muscle thickness in growing children with Class II malocclusion is independent of sex but correlated with age, with older patients presenting thicker masseter muscles.
- In children with thicker masseter muscles, smaller intermaxillary and gonial angles respectively were found.

## CONFLICT OF INTERESTS

The authors declare that there are no conflicts of interests.

## AUTHOR CONTRIBUTIONS

Gregory S. Antonarakis and Stavros Kiliaridis conceived, designed, and supervised the study. Eirini Tentolouri and Ioanna Georgiakaki conducted sample collection and analysis. Gregory S. Antonarakis carried out the statistical elaboration of the project. Eirini Tentolouri, Gregory S. Antonarakis, and Stavros Kiliaridis contributed to the interpretation of the data and writing of the manuscript. All authors read and approved the final manuscript.

## Data Availability

The data that support the findings of this study are available from the corresponding author upon reasonable request.
